# Effects of Capsicum Oleoresin Supplementation on Lactation Performance, Plasma Metabolites, and Nutrient Digestibility of Heat Stressed Dairy Cow

**DOI:** 10.3390/ani12060797

**Published:** 2022-03-21

**Authors:** Zhigao An, Xinxin Zhang, Shanshan Gao, Di Zhou, Umair Riaz, Mohamed Abdelrahman, Guohua Hua, Liguo Yang

**Affiliations:** 1 National Center for International Research on Animal Genetics, Breeding and Reproduction (NCIRAGBR), College of Animal Science and Technology, Huazhong Agricultural University, Wuhan 430070, China; anzhigao95@foxmail.com (Z.A.); zxx1144795936@163.com (X.Z.); 17638563283@163.com (S.G.); hzau_judy_213@163.com (D.Z.); umair.riaz@iub.edu.pk (U.R.); mohamed.asad@agr.au.edu.eg (M.A.); huaguohua@mail.hzau.edu.cn (G.H.); 2 Faculty of Veterinary and Animal Sciences, The Islamia University of Bahawalpur, Bahawalpur 63100, Pakistan; 3 Animal Production Department, Faculty of Agriculture, Assuit University, Asyut 71515, Egypt; 4 Hubei Engineering Research Center in Buffalo Breeding and Products, Wuhan 430070, China

**Keywords:** Capsicum oleoresin, milk production, heat stress alleviation, blood physiology

## Abstract

**Simple Summary:**

Heat stress has significant adverse effects on the lactation performance of dairy cows. Therefore, novel feed additives that alleviate heat stress are continuously being researched and developed to deal with this predicament. On this basis, the effects of Capsicum oleoresin supplementation on the lactation performance, rectal temperature, plasma metabolites, and nutrient digestibility of dairy cows were studied. The results showed that the dietary addition of Capsicum oleoresin could improve dry matter intake, milk yield, and milk quality, and reduce the rectal temperature of dairy cows. Furthermore, Capsicum oleoresin supplementation also alters plasma metabolites. In summary, Capsicum oleoresin could effectively be used as a feed additive to relieve heat stress in dairy cows.

**Abstract:**

The present study investigates the effect of Capsicum oleoresin (CAP) supplementation on the dry matter intake, milk performance, plasma metabolites, and nutrient digestibility of dairy cows during the summer. Thirty-two lactating Holstein dairy cows (*n* = 32) were randomly divided into four groups. The CAP was dissolved in water and added to the total mixed ration with graded levels of CAP (0, 20, 40, and 80 mg/kg of dry matter). The trial period consisted of seven days for adaptation and thirty days for sampling. Data were analyzed using the MIXED and GLM procedure SAS. The linear and quadratic effects were tested. The milk yield, milk fat, and milk urea nitrogen increased linearly with the dietary addition of CAP (*p* < 0.05). The dry matter intake increased linearly in the 20CAP group (*p* < 0.05). Additionally, the 4% fat-corrected milk, energy-corrected milk, milk fat yield, and milk fat to milk protein ratio increased quadratically (*p* < 0.05), while the rectal temperature decreased quadratically (*p* < 0.05). Serum total cholesterol and non-esterified fatty acids increased linearly (*p* < 0.05); glucose and β-hydroxybutyrate tended to increase quadratically with the dietary addition of CAP (*p* = 0.05). Meanwhile, CAP supplementation did not affect the milk protein yield, blood concentration of triglyceride, insulin, lipopolysaccharide, immunoglobulin G, or heat shock protein 70 expression level (*p* > 0.05). In addition, nutrient digestibility was comparable among groups (*p* > 0.05). These findings indicated that CAP supplementation could enhance the lactation performance of dairy cows during the summer.

## 1. Introduction

Heat stress is one of the most critical environmental stressors during hot and humid environments in the dairy cattle industry [1]. Previous studies have reported the negative effect of heat stress on the health and lactation performance of dairy cows [2,3,4]. In terms of milk yield, heat stress is primarily responsible for a reduction in milk production of approximately 15–27.6% [5,6]. The dairy industry loses 1.2 billion USD per year due to heat stress and consequent decreased milk production from lactating cows in the USA, and global climate change increases economic losses [7]. In addition, heat stress negatively affects the lipid metabolism in dairy cows, leading to an increased incidence of nutrition-related metabolic diseases [8]. The temperature–humidity index (THI) is the indicator of environmental parameters, in which dairy cows live [9]. Lactating dairy cows enter a mild state of heat stress when THI reaches over 72 [10]. Although some cooling systems (shade, fans, sprinklers, etc.) at dairy farms partially alleviate heat stress [11,12], new strategies are still needed to maintain dairy cows’ physiological, metabolic adaptation and milk yield performance.

Capsaicin (*trans*-8-methyl-N-vanillyl-6-noneneamide) is the source of spicy-aromatic compounds in the capsicum genus and the active ingredients in Capsicum oleoresin. Dietary capsaicin can increase food intake by directly acting on the neurons [13] and stimulate the secretion of digestive juices [14] in the digestive tract. The dietary intake of capsaicin reportedly improved feed efficiency in chickens [15], pigs [16,17], goats [18], sheep [19], beef [20], and dairy cows [21,22,23]. Capsaicin supplementation could improve milk production as a result of enhanced fat metabolism in dairy cows [22]. Meanwhile, capsaicin could be involved in body temperature regulation. Vasodilation and increased blood flow were reported as potential effects on account of the stimulating neurons by capsaicin [24,25].

The present study hypothesized that CAP may: (1) increase feed intake; (2) increase lipid mobilization and the glucose level in plasma; (3) decrease the rectal temperature; (4) improve lactation performance; and (5) positively affect nutrient digestibility. Therefore, the specific intentions of the present study are to understand the potential effects of CAP on blood chemistry, milk performance, and rectal temperature in dairy cows under heat stress.

## 2. Materials and Methods

### 2.1. Animals and Experimental Design

This experiment was conducted at a commercial dairy farm in Hubei, China, in July 2019. Thirty-two lactation Holstein cows (milk yield, 26.0 ± 2.60 kg/d; DIM, 150 ± 20.3 d; and body weight, 657 ± 93.3 kg, at the beginning of the experiment) were enrolled in this experiment and randomly divided into four groups (*n* = 8/group). The experiment lasted thirty days after seven days of CAP adaptation. The four experimental groups included a control group (0 mg CAP/kg dry matter) and three treatment groups, which were given 20, 40, and 80 mg CAP/kg of dry matter (DM). The CAP (MY1098, 10.0% capsaicin; Tianxu Food Additive Co., Ltd., Guangzhou, China) was dissolved in water and added to the total mixed ration (TMR) before feeding. The TMR was formulated according to NRC [26], and the ingredients and nutritional level are shown in Table 1. All the cows were fenced into four groups and housed with sprinklers and two rows of fans in the barn. The fans were activated throughout the experiment. When the ambient temperature reached 22 °C, the sprinklers were activated for 40 s every 5 min. Cows were kept in line with the same cooling system, and had free access to freshwater and a diet with 10% refusal. The cows were milked twice a day at 12-h intervals (5 a.m. and 5 p.m.).

### 2.2. Sampling and Analyses

The individual dry matter intake (DMI) was measured by weighting the feed offered and refused once a week, and the dietary sample was collected weekly and frozen at −20 °C. Then, it was dried for 48 h at 65 °C and crushed, through a 1 mm sieve for use in the analysis of dry matter (DM) ([27]; method 945.15), crude protein (CP) ([27]; method 984.13), ether extract (EE) ([27]; method 920.29), ash ([27]; method 942.05), and acid-insoluble ash (AIA) [28]. The content of neutral detergent fiber (NDF) (using α-amylase and sodium sulfite) and acid detergent fiber (ADF) was analyzed, as described by Van Soest et al. [29]. Calcium and phosphorus were determined using atomic absorption spectroscopy [30] and spectrophotometry ([27]; method 991.25), respectively. Soluble-carbohydrate was analyzed as described in the work of McDonald and Henderson [31].

The temperature and relative humidity were recorded three times a day (8 a.m., 2 p.m., and 8 p.m.) using a temperature and humidity recorder (COS–04, Peoplesoft Measurement and Control Technology Co., Ltd., Shandong, China), and the THI was calculated using the method of NRC [32]:THI = (1.8 × temperature °C + 32) − [(0.55 − 0.0055 × relative humidity %) × (1.8 × temperature °C − 26)]. The rectal temperature (RT) was measured at 2 p.m. using a mercury thermometer at the end of the experiment. The milk yield was measured weekly. Individual milk samples (50 mL) were collected at 5 a.m. and 5 p.m., mixed according to the actual production by volume at the end of the experiment and preserved by adding potassium dichromate. The milk composition was measured using a milk composition analyzer (CombiFoss FT+, Shanghai Jinmai Instrument Equipment Co., Ltd., Shanghai, China) for dairy herd improvement (DHI) in Hubei.

The fecal samples (200 g) were collected individually from the rectum in the final week for 3 days every 8 h [33], then mixed with feces from each dairy cow and stored at −20 °C. Nitrogen was fixated by 10% 3 mol/L sulfuric acid based on the weight after the collection. Then, it was dried at 65 °C, crushed, and passed through a 1 mm sieve to measure organic matter (OM), CP, NDF, ADF, and AIA, as indicated above. AIA was used as an internal marker to estimate nutrient digestibility based on the concentration of AIA in the feed and feces [34].

At 2 p.m., blood serum was collected via the coccygeal vein of d 30, centrifuged for 15 min at 3000× *g* at room temperature and preserved at −20 °C until further analysis. The serum was analyzed for glucose (Glu) and total cholesterol (TC) (Bs-420 Automatic Biochemical Analyzers, Mindray Biomedical Electronics Co., Ltd., Shenzhen, China). Moreover, serum was used for β-hydroxybutyric acid (β-HB), immunoglobulin G (IgG), triglyceride (TG), non-esterified fatty acid (NEFA), heat shock protein 70 (HSP-70), and insulin measurements using anELISA kit (Jiyinmei Technology Co., Ltd., Wuhan, China)

### 2.3. Statistical Analysis

Measurements of DMI, milk yield, and milk composition were analyzed with repeated measures using the Statistical Analysis System (SAS, 2005). The DMI records, milk yield, and milk composition were measured before the experiment and were saved as covariates and autoregressive in the covariance structure, using PROC MIXED. The fixed effects in the model included the treatment, time, and treatment × time interaction. The variance for each cow was used as the random effect. Data for RT, digestibility, and plasma measurements were analyzed using PROC GLM. All data are expressed as covariate-adjusted least squares means. Duncan’s multiple range test was used to test the significance of differences among means, and orthogonal polynomial contrasts were used to further divide the repetition effects into linear and quadratic effects. Standard errors of the mean were reported, and differences between treatments were considered statistically at *p* < 0.05 and tendency at 0.05 ≤ *p* < 0.10.

## 3. Results

### 3.1. THI and Determination of Heat Stress

The mean of THI (Table 2) was 77.8 to 83.9 during the experimental period. This indicated that dairy cows were under moderate heat stress for the most part based on THI levels (72 ≤ THI < 82; [10,35]). The average RT of dairy cows, as shown in Table 3, was 39.99 ± 0.24 °C in the control group. Therefore, it was suggested that dairy cows in each group were heat stressed during the experiment. The RT of dairy cows decreased quadratically with the dietary addition of CAP under heat stress, which was the lowest at 39.25 ± 0.28 °C in the 20 CAP group (*p* < 0.05).

### 3.2. DMI

**Figure 1:**
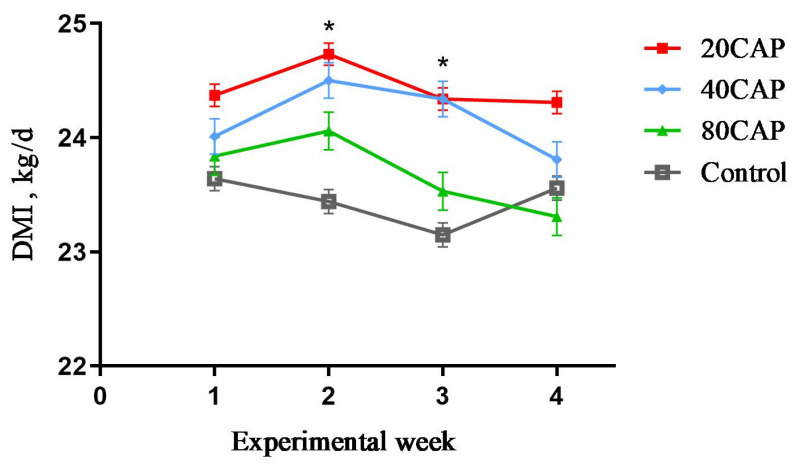
Change in average dry matter intake (DMI) throughout the study period in dairy cows that were fed a basal diet (control) or a basal diet supplemented with 20 (20CAP), 40 (40CAP), or 80 (80CAP) mg of CAP/kg of dry matter. Bars indicate the standard errors. Linear effect, *p* = 0.01; week effect, *p* < 0.01; week x treatment effect, *p* = 0.24. * indicates linear effect (*p* < 0.05).

The result of DMI by supplementation of CAP is shown in Table 3. In the 20CAP group, the DMI increased linearly (*p* < 0.05). The change in DMI during the experimental period is shown in Figure 1.

### 3.3. Milk Yield and Milk Composition

**Figure 2:**
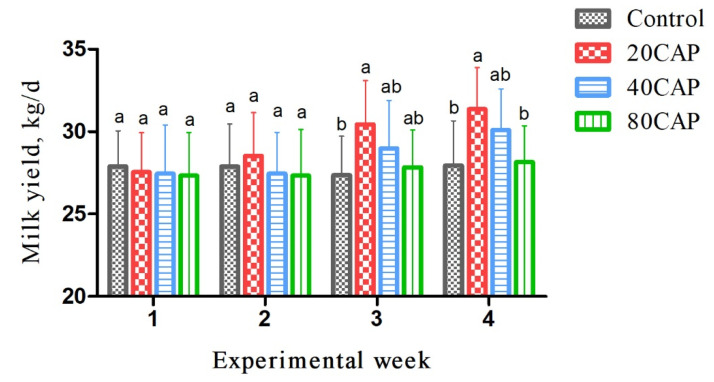
Change in milk yield throughout the study for cows fed a basal diet (control) or basal diet supplemented with 20 (20CAP), 40 (40CAP), or 80 (80CAP) mg of CAP/kg of dry matter. Bars indicate the standard error. Different letters (a, b) indicate the significant difference (*p* < 0.05).

The milk yield in cows supplemented with 20 mg/kg of DM was higher than that of the control and other treatment groups during the experiment, as presented in Figure 2. The milk yield increased linearly (*p* < 0.05) with the increasing addition of CAP. The diet addition of CAP linearly increased the milk fat and milk urea nitrogen (MUN) (*p* < 0.05). A significant quadratic effect was detected for 4% fat corrected milk (4%FCM) and energy-corrected milk (ECM), whereby a maximum was reached under the 20CAP treatment (*p* < 0.05). In addition, CAP quadratically increased milk fat yield and the fat to protein ratio, which was highest in the 40CAP treatment (*p* < 0.05). There was no effect on milk protein and milk protein yield with the increasing addition of CAP (Table 3).

### 3.4. Blood Indicators

As shown in (Table 4), the blood indicators Glu and β-HB had a quadratic trend with CAP addition (*p* = 0.05). The concentrations of NEFA were decreased linearly (*p* < 0.05) with increasing levels of CAP. The concentrations of TC linearly increased with the increasing addition of CAP (*p* < 0.05). There was no difference in TG, insulin, LPS, IgG, and HSP-70 with the increasing addition of CAP.

### 3.5. Diet Nutrient Digestibility

The apparent digestibility of DM, OM, CP, NDF, and ADF showed no significant variation in *p* > 0.05 among groups regarding the dietary addition of CAP (Table 5).

## 4. Discussion

Heat stress is a great challenge for the global dairy industry, especially at low latitudes with long periods of hot weather. RT increases with increased environment temperature during heat stress and negatively correlates with DMI in dairy cows [36,37,38]. In this study, RT decreased quadratically with the increasing addition of CAP in the diet, while the average THI reached 77.8–83.9. It has been suggested that capsaicin causes hypothermia via heat loss, increased skin surface temperature or perspiration in mice and rats, and in humans via oral intake [39,40,41]. The quadratic decrease in RT in this experiment may have a similar reason, despite the fact that HSP-70 did not differ between groups. Unfortunately, we did not measure the surface temperature of dairy cows.

In the present study, DMI increased linearly in the 20CAP group, which increased the energy intake of dairy cows. It is probable that the decrease in RT leads to a linear increase in the DMI of the 20CAP group due to heat stress moderated by CAP in this research. On the other hand, high concentrations of CAP reduced the DMI and eating speed in dairy cows due to its irritation; meanwhile, CAP may partially degrade in the rumen [20,42,43]. Therefore, there was no RT drop-off with a high CAP diet concentration in dairy cows, while RT was increased.

The present study showed a linear increase in milk yield, yet a quadratic increase in ECM and 4%FCM, as a result of the dietary addition of CAP. This seems to be due to the decline in RT and change in DMI that caused the relief of heat stress and restriction in energy intake. The dietary addition of CAP increased linearly for the milk fat and quadratically for the milk fat yield, while milk protein was not affected, which is different from a previous study of dairy cows [44]. Meanwhile, the fat-to-protein ratio of milk showed a linear increase with the addition of CAP, similar to that of milk fat. It has been documented that there is a decrease in milk protein concentration, although no effect on milk fat in heat stress [45].

Contrary to previous studies [46], dietary CAP quadratically increased glucose in blood, while insulin was not affected, which reported that the triglyceride mobilization blocked with insulin was increased and the NEFA was unvaried at low glucose levels under heat stress [37]. In this experiment, NEFA decreased linearly and β-HB increased quadratically with the addition of CAP, indicating that the energy dilemma was improved due to CAP. Furthermore, some studies have suggested that capsaicin increased lipolysis and mitigated the adverse effects of NEB by increasing the oxidation of fat [47,48]. Thus, CAP promoted the NEFA oxidation and led to a quadratic increase in glucose and β-HB in serum in the present study. TG did not change in the same context as the previous study [49]. Therefore, it seems that the dietary addition of CAP may have promising potential for improving energy intake and use among heat-stressed dairy cows. Additionally, the linear increase in MUN was due to the change in DMI.

There was no significant variation in IgG and LPS in a different group of CAP addition in the present study. Several studies have revealed that heat stress increased intestinal permeability [50,51], despite some studies not supporting these findings [52]. Additionally, it is widely believed that rumen barrier function to increase energy intake is impaired under high concentrate diets [53,54]. It is a common nutritional strategy employed during heat stress, including in the present study. These factors all add to the adverse effects of heat stress. This research suggests that other factors may influence IgG or LPS entering the blood from other sites in the alimentary canal.

In the present study, there was no difference in the apparent digestibility among the CAP groups. This is similar to previous research [49]. Previous studies reported that rumen fermentation was altered and the acetate-to-propionate ratio was reduced [44], and CAP increased total volatile fatty acids in dairy cows [23]. Meanwhile, capsaicin can be used as an antimicrobial agent on account of its inhibitory effect on a variety of bacteria [55,56]. Thus, it was indicated that there are no adverse effects on nutrient digestibility based on the dietary addition of CAP.

## 5. Conclusions

There were beneficial effects on dry matter intake, rectal temperature, and milk performance due to the dietary supplementation of CAP in the present study. During the summer, the optimal CAP additive amount was 20 mg/kg of dry matter for dairy cows in this research.

## Figures and Tables

**Table 1:** Ingredient and chemical composition of the diet during the experiment (DM basis).

Ingredients	Content (%)	Nutritional Level	Content (%)
Corn silage	19.63	CP	16.79
Alfalfa hay	16.80	NDF	31.48
Cottonseed, whole	6.28	ADF	11.32
Soybean hulls, pelleted	4.29	EE	8.35
Oat hay	4.27	Soluble-carbohydrate	3.94
Soybean meal	4.17	Ash	7.72
Commercial concentrate mixture ^1^	43.38	Ca	0.80
Sodium bicarbonate	1.18	P	0.77

^1^ The commercial concentrate mixture (CJ Feed Co., Ltd., Zhengzhou, China) contained 18.72% CP, 17.31% EE, 23.77% NDF, 6.34% ADF, 0.06% ash on DM basis.

**Table 2:** Average and range weekly values for air temperature (T), relative humidity (RH), and temperature–humidity index (THI).

	Experimental Week							
	1		2		3		4	
Variable	Mean	Range	Mean	Range	Mean	Range	Mean	Range
T (°C)	27.2	22.1–33.5	28.1	23.4–33.5	27.1	22.3–33.6	30.6	27.5–35.4
RH (%)	78.6	56.5–97.5	81.6	52.6–90.8	78.9	41.2–93.0	81.1	63.3–90.5
THI ^1^	78.0	70.6–86.9	79.9	73.4–83.3	77.8	71.6–84.4	83.9	77.5–88.3

^1^ THI was calculated by the method of NRC [32]: THI = (1.8 × temperature °C + 32) − [(0.55 − 0.0055 × relative humidity %) × (1.8 × temperature °C − 26)].

**Table 3:** Effects of Capsicum oleoresin (CAP) on production variables in heat-stressed dairy cows.

Parameter	Treatment ^1^					*p*-Value ^2^	
	Control	20CAP	40CAP	80CAP	SEM	L	Q
DMI, kg/d	23.26 ^b^	24.43 ^a^	23.90 ^ab^	23.45 ^ab^	0.21	0.01	0.13
RT, °C	39.99	39.25	39.84	39.94	0.19	0.49	0.03
Milk yield							
Milk, kg/d	27.14 ^ab^	30.73 ^a^	29.46 ^ab^	28.32 ^b^	2.41	0.01	0.08
4%FCM ^3^, kg/d	29.17	32.83	31.58	29.72	4.72	0.60	0.02
ECM ^4^, kg/d	31.36	35.39	33.68	32.32	4.89	0.39	0.02
Milk solids concentration							
Fat, %	4.61 ^ab^	4.28 ^ab^	4.99 ^a^	4.01 ^b^	0.66	0.03	0.18
Protein, %	3.45	3.43	3.45	3.39	0.18	0.16	0.57
MUN, mg/dL	10.84 ^b^	15.33 ^ab^	15.05 ^ab^	16.78 ^a^	3.13	0.01	0.25
Fat/Protein	1.29	1.31	1.44	1.18	0.13	0.09	0.01
Milk solids yields							
Fat, kg/d	1.24	1.34	1.37	1.18	0.21	0.44	0.02
Protein, kg/d	1.24	1.34	1.37	1.18	0.12	0.20	0.47

^a,b^ Means within a row with unlike superscripts differ (*p* < 0.05). ^1^ Cows were fed a basal diet (control) or basal diet supplemented with 20, 40, or 80 mg of CAP/kg of DM. ^2^ L = linear; Q = quadratic. ^3^ 4%FCM = 0.4 (kg of milk) + 15.0 (kg of fat). ^4^ ECM = 0.327 × milk (kg) + 12.95 × fat (kg) + 7.20 × protein (kg).

**Table 4:** Effects of Capsicum oleoresin (CAP) on blood metabolites in heat-stressed dairy cows.

Parameter	Treatment ^1^					*p*-Value ^2^	
	Control	20CAP	40CAP	80CAP	SEM	L	Q
Glu, mmol/L	4.44	4.76	5.30	4.65	0.23	0.28	0.05
TG, mmol/L	6.71	6.47	6.62	6.94	0.28	0.51	0.36
TC, mmol/L	5.19 ^ab^	4.93 ^b^	5.38 ^a^	5.33 ^a^	0.08	0.03	0.26
β-HB, μmol/L	8.89	10.47	10.13	9.99	0.40	0.11	0.05
NEFA, ng/mL	237.38 ^a^	215.91 ^ab^	213.58 ^ab^	200.12 ^b^	7.05	0.01	0.95
Insulin, mU/L	43.12	44.79	41.40	41.18	1.54	0.57	0.70
LPS, ng/mL	90.82	79.90	92.36	91.12	5.10	0.57	0.38
IgG, μg/mL	20.49	21.88	21.54	21.60	0.80	0.68	0.63
HSP-70, ng/mL	45.44	42.28	45.97	46.64	1.68	0.35	0.29

^a,b^ Means within a row with unlike superscripts differ (*p* < 0.05). ^1^ Cows were fed a basal diet (control) or basal diet supplemented with 20, 40, or 80 mg of CAP/kg of DM. ^2^ L = linear; Q = quadratic.

**Table 5:** Effects of Capsicum oleoresin (CAP) on apparent total tract digestibility of nutrients in heat-stressed dairy cows.

		Treatment ^1^				*p*-Value ^2^	
Apparent Digestibility (%)	Control	20CAP	40CAP	80CAP	SEM	L	Q
DM	73.76	74.71	72.39	76.09	0.03	0.60	0.50
OM	75.79	76.49	74.26	78.03	0.03	0.61	0.44
CP	74.51	76.63	74.66	76.82	0.03	0.56	0.98
NDF	69.02	70.41	66.72	74.16	0.04	0.27	0.22
ADF	61.63	62.42	58.54	64.40	0.05	0.75	0.42

^1^ Cows were fed a basal diet (control) or basal diet supplemented with 20, 40, or 80 mg of CAP/kg of DM. ^2^ L = linear; Q = quadratic.

## Data Availability

The datasets used and/or analyzed during the current study are available from the corresponding author upon reasonable request.
